# *N*-Glycans from Porcine Trachea and Lung: Predominant NeuAcα2-6Gal Could Be a Selective Pressure for Influenza Variants in Favor of Human-Type Receptor

**DOI:** 10.1371/journal.pone.0016302

**Published:** 2011-02-09

**Authors:** Nongluk Sriwilaijaroen, Sachiko Kondo, Hirokazu Yagi, Nobuhiro Takemae, Takehiko Saito, Hiroaki Hiramatsu, Koichi Kato, Yasuo Suzuki

**Affiliations:** 1 Faculty of Medicine, Thammasat University (Rangsit Campus), Pathumthani, Thailand; 2 Health Science Hills, College of Life and Health Sciences, Chubu University, Kasugai, Aichi, Japan; 3 Graduate School of Pharmaceutical Sciences, Nagoya City University, Aichi, Japan; 4 GLYENCE Co., Ltd., Aichi, Japan; 5 Thailand-Japan Zoonotic Diseases Collaboration Center, Chatuchak, Bangkok, Thailand; 6 Research Team for Zoonotic Diseases, National Institute of Animal Health, National Agriculture and Food Research Organization (NARO), Tsukuba, Ibaraki, Japan; 7 Institute for Molecular Science and Okazaki Institute for Integrative Bioscience, National Institutes of National Sciences, Aichi, Japan; 8 The Glycoscience Institute, Ochanomizu University, Tokyo, Japan; 9 Global COE Program for Innovation in Human Sciences, University of Shizuoka School of Pharmaceutical Sciences, Shizuoka, Japan; Centers for Disease Control and Prevention, United States of America

## Abstract

It is known that pigs acted as “mixing vessels” for genesis of a new reassortant influenza strain responsible for pandemic H1N1 2009. However, the host factors driving the evolution of a reassorted virus in pigs to ‘jump species’ resulting in a human outbreak remain unclear. *N*-glycans derived from the porcine respiratory tract were enzymatically released, fluorescent labeled with 2-aminopyridine, separated according to charge, size and hydrophobicity, and structurally identified by a two-dimensional (size and hydrophobicity) HPLC mapping technique and MALDI-TOF mass spectrometry before and after exo-glycosidase digestion. We found a 3-, 5-, and 13-fold increases in NeuAcα2-6, a preferable human influenza receptor, over NeuAcα2-3, an avian influenza receptor, from upper and lower parts of the porcine trachea towards the porcine lung, a major target organ for swine virus replication. The large proportion of NeuAcα2-6 may exert selective pressure for selection of influenza variants with altered receptor preference for this human-type α2-6 receptor, a crucial first step for generating a human pandemic.

## Introduction

Influenza A viruses cause local epidemics every year and occasionally cause worldwide pandemics, which have been considered to be major public health threats. Viruses in pandemic outbreaks acquire mutations that evade human immunity and efficiently transmit from human to human. Viruses carrying a hemagglutinin (HA) surface glycoprotein to which humans are immunologically naive can be derived from an avian virus or an avian-human reassortant virus [1]. Although there have been many reports on direct transfer of an avian virus, especially highly pathogenic H5N1, to humans [2], [3], transmission between humans of avian viruses has been limited, inefficient and unsustained [4]. A number of factors, including the RNA polymerase PB2 subunit and HA activation proteases, may be involved in host range restriction and pathogenicity of influenza viruses [1], [5]; however, HA plays a key role in the initial stage of infection and thus functions as a critical host range determinant [6]. HA recognizes host glycans with terminal sialic acids (Sia), which vary in structure among species and their tissues. Avian HA prefers host glycans with Siaα2-3Gal linkage, mainly found on the bird intestine and respiratory epithelia, whereas human HA prefers those with the Siaα2-6Gal linkage, which populate the human upper respiratory epithelia [7], a major site of its infection in humans [8]. To overcome this interspecies barrier, avian HA must switch its binding preference to Siaα2-6Gal. Three historical influenza pandemics, Spanish H1N1 in 1918, Asian H2N2 in 1957 and Hong Kong H3N2 in 1968 as well as the present pandemic H1N1 2009 have HAs with preferential recognition of Siaα2-6Gal, even though their HAs are of non-human origin [9]. Although it remains a mystery whether the 1918 pandemic virus was transmitted directly from avians or was the result of reassortment before the pandemic [10], [11], the 1957, 1968 and 2009 pandemic viruses were reassortant viruses [12], and the 2009 pandemic was confirmed to be of swine origin and was hence named swine-origin H1N1 influenza viruses (S-OIVs) [12]. Although S-OIV causes mild disease, it has spread worldwide to more than 214 countries and has caused over 18,449 laboratory-confirmed deaths as of August 1, 2010 [13] due to relatively efficient transmission among humans.

Swines susceptible to both avian and human influenza viruses and possessing a trachea that expresses both Siaα2-3Gal (avian receptor) and Siaα2-6Gal (human receptor) [14] provide direct evidence supporting the theory of pigs as “mixing vessels” for the creation of reassortant viruses. However, receptor binding specificity data have shown that classical swines preferentially recognized NeuAcα2-6Gal [14], [15], [16], [17] and that avian-like swine switched their binding preference to NeuAcα2-6Gal over time [14], [15], [16]. Analysis of amino acids has suggested that HA mutations are responsible for the increase in affinity of the virus for NeuAcα2-6Gal [14], [15], [16]. However, why influenza viruses that have continued replication in pigs evolve HA receptor specificity to NeuAcα2-6Gal is still puzzling. Types, structures and distribution of glycans on the host cell surface are thought to be associated with viral HA receptor specificity. Thus, we elucidated the structures of *N*-glycans required for influenza A infection [18] from the porcine trachea, an early site of influenza virus attack and replication, and porcine lung, a principal swine influenza replication site [19], by HPLC and matrix-assisted laser desorption/ionization time-of-flight mass spectrometry (MALDI-TOF-MS) analyses.

## Materials and Methods

### Ethics statement

Animal experiments were carried out according to the regulations and guidelines approved by the Animal Ethics Committee of the National Institute of Animal Health, Japan. The approved number is 08-058.

### Porcine trachea and lungs

Porcine trachea and lungs were collected from a clinically healthy 5-year-old female LWD-pig, an offspring of a male Duroc (D) and a female F1 hybrid pig (a female Landrace (L) and a male Large White (W)).

### Lectin detection of Siaα2-3Gal and Siaα2-6Gal receptors

The porcine trachea, which was divided into upper and lower parts, and the lungs were rinsed with ice-cold phosphate-buffered saline (PBS, pH 7.2) and then cut into 3-mm^3^ cubes. Tissue blocks were fixed in 4% paraformaldehyde (PFA) in PBS overnight at 4°C and equilibrated in 30% sucrose in PBS overnight at 4°C. The blocks were embedded in OCT compound (Sakura Finetechnical Co. Ltd., Japan) and frozen at −80°C for cryosectioning. Cryostat sections (10 µm each) were air-dried and re-hydrated twice in PBS containing 0.1% Tween-20 (PBST) for 5 min each time at room temperature. Immunostaining was begun by adding 50 µl of a lectin mixture of biotin-conjugated *Sambucus niga* agglutinin (SNA; specific for NeuAcα2-6Gal; 5 µg/mL; Vector Lab, Burlingame, CA) and digoxigenin (DIG)-conjugated *Maackia amurensis* agglutinin (MAA; specific for NeuAcα2-3Gal; 10 µg/mL; Roche, Mannheim, Germany) to PBS containing 0.05% Tween-20 and 0.5% bovine serum albumin (BSA). After incubation for 30 min at 37°C, the sections were washed three times with PBST for 5 min each time. The sections were incubated with 50 µl of a solution of fluorescein-conjugated avidin (Zymed, USA) and rhodamine-conjugated anti-DIG antibodies (Roche) at a dilution of 1∶100 for 30 min at 37°C. After three washes with PBST, stained sections were mounted on slides in buffered glycerol (pH 9.0) and examined using a fluorescence microscope (Olympus B1, Tokyo, Japan). Negative controls were done in the absence of lectin.

### Preparation of porcine trachea and lungs for *N*-glycan analysis

The porcine tracheal tube was cut transversely into upper (14.14 g) and lower (10.48 g) parts. Each part was opened longitudinally, and its luminal surface was cleaned with cold 0.9% NaCl, removed by scraping, and cut into small pieces. The pieces were pooled, lyophilized (400 mg for the upper part and 560 mg for the lower part), and kept at -80°C until use.

The porcine lung (6,225 mg) was cleaned and cut into small pieces and then homogenized using a T10 Basic Ultra-Turrax homogenizer (IKA-Werke, Germany) followed by a glass-Teflon homogenizer (Eyela, Tokyo, Japan) in a cold buffer containing 20 mM Tris-HCl (pH 7.6), 150 mM NaCl, 0.3 M sucrose and protease inhibitor cocktail set 1 (Calbiochem, Sandiego, CA). The homogenate was then centrifuged at 1000 *g* for 10 min and the post-nuclear supernatant was ultracentrifuged at 100,000 *g* for 1 h (Beckman, SW28 rotor). The pellet (membrane fraction) was resuspended in 0.9% NaCl and recentrifuged. The washed pellet was dried by a lyophilizer (125 mg).

### Isolation and identification of *N*-glycan structures

The lyophilized porcine trachea (upper and lower parts) and lungs were delipidated by extraction solvents, 80% ethanol (three times), 100% ethanol, chloroform/methanol (2∶1,v/v) and chloroform/methanol/H_2_O (1∶2∶0.8, v/v/v), in that order and used as the starting material for structural analysis. The conditions for enzymatic release, fluorescent labeling, separation and structural identification of *N*-glycans were the same as those reported previously [20], [21], [22]. In brief, the delipidated extract (20 mg) was proteolyzed with pepsin plus glycoamidase A and the resultant peptides were further digested with pronase to amino acids. The reducing ends of the released *N*-glycans after purification by Bio-Gel P-2 chromatography were labeled with fluorescent 2-aminopyridine. After removal of excess reagents by Sephadex G-15 chromatography, the pyrimidylamino (PA)-glycans were separated by a TSK-gel diethylaminoethyl (DEAE)-5PW column (Tosoh, Tokyo) based on charge. Each separated fraction was further applied to a Shim-pack HRC-octadecyl silica (ODS) column (Shimadzu, Kyoto) for separation on the basis of hydrophobicity, and the elution time of each peak was expressed as glucose unit (GU) value. The molecular mass of each PA-glycan fraction was analyzed by MALDI-TOF-MS. Fractions containing two kinds of glycans were further subfractionated according to molecular sized by a TSK-gel Amide-80 column (Tosoh) and the elution positions were calibrated in GU values. Sample PA-glycans were mapped on the basis of on their GU and molecular mass values and their coordinates were compared with those of reference PA-glycans in the GALAXY database [23]. Sample PA-glycans showing no agreement with any PA-glycans in the database were sequentially trimmed with exoglycosidases: α-galactosidase (coffee bean, Oxford GlycoScience, Bedford, MA); β-galactosidase (jack bean, Seikagaku Kogyo, Tokyo, Japan); α-sialidase (*Arthrobacter ureafaciens*, Nacalai Tesque, Kyoto, Japan); and α2,3-sialidase (*Salmonella typhimurium* LT2, Takara Bio Inc, Otsu, Japan) under conditions described previously [21], [22], [24]. Each trimmed PA-glycan at each step was analyzed until its elution and mass coordinates became identical with the known reference on the map. The structures were confirmed by co-chromatography of the sample and reference PA-glycans.

## Results

### Distribution of Siaα2-3Gal and Siaα2-6Gal receptors in the porcine trachea (upper and lower parts) and lungs

**Figure 1:**
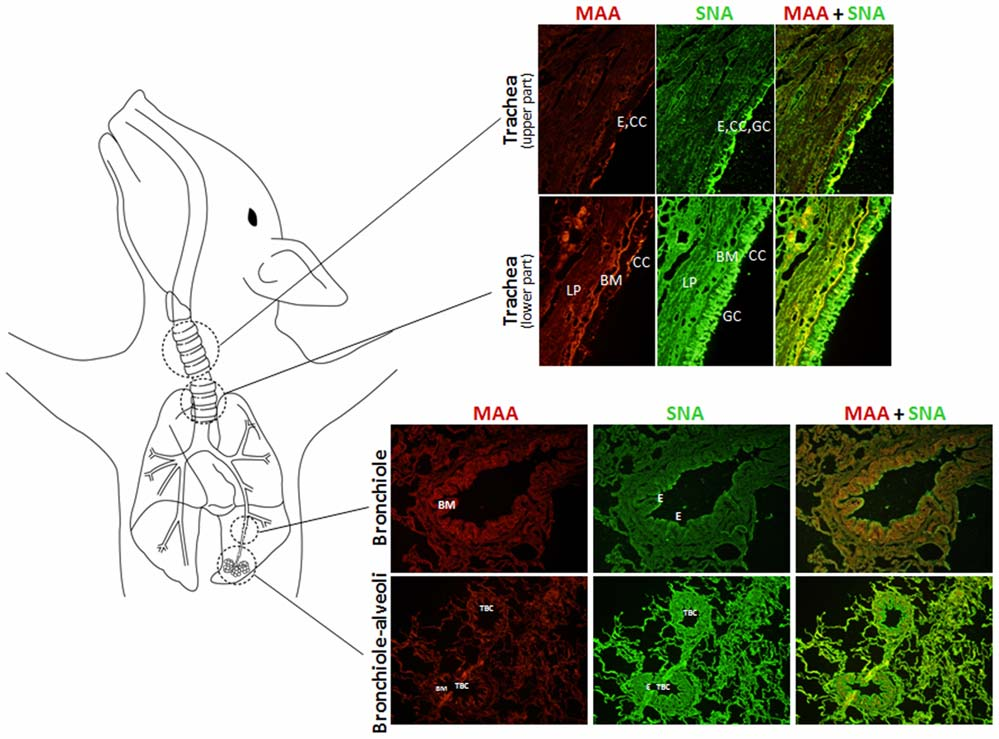
Distribution of Siaα2-3Gal and Siaα2-6Gal receptors in the porcine respiratory tract detected by lectin staining. Sections containing Siaα2-3Gal and Siaα2-6Gal receptors were reacted with DIG-labeled MAA and biotinylated SNA, respectively. DIG-MAA- and biotin-SNA-exposed sections were then reacted with anti-DIG-rhodamine (red) and avidin-fluorescein (green), respectively. Sections from representative tissues are shown at original magnification of x400. E =  epithelium, CC  =  ciliated cells, GC  =  goblet cells, BM  =  basement membrane, LP  =  lamina propria, TBC  =  terminal bronchiole.

Double-labeling of sections of the porcine trachea and lungs (Figure 1) revealed that both MAA lectin, specific for Siaα2-3Gal linkages, and SNA lectin, specific for Siaα2-6Gal linkages, were stained in both the upper and lower parts of the trachea and in the lung with SNA binding being more dominant than MAA binding. On the ciliated pseudostratified columnar epithelium of the upper and lower parts of the trachea, MAA reacted predominantly with ciliated cells, whereas SNA also reacted with goblet cells. Both bronchiole and alveoli were stained by SNA and MAA. However, SNA was observed as a fine line at the bronchial epithelial border, whereas MAA was not.

### *N*-glycans of the porcine trachea (upper and lower parts) and lungs

**Figure 2:**
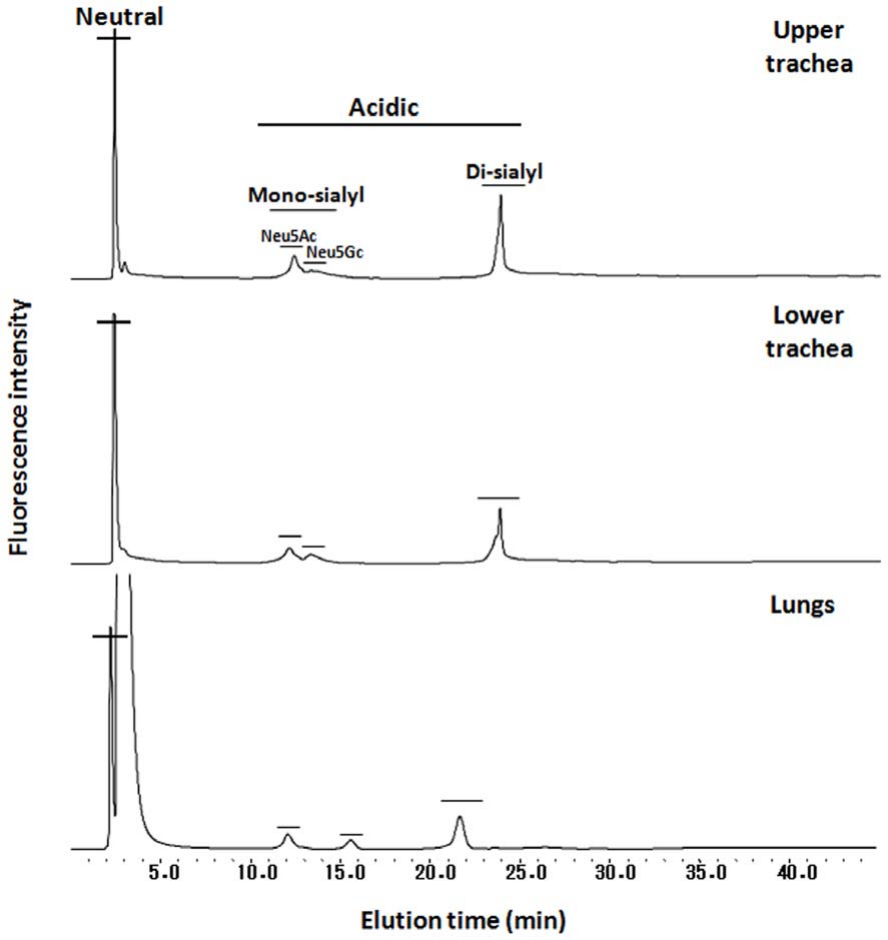
Anion exchange DEAE elution profiles of PA-glycans derived from the porcine trachea (upper and lower) and lungs. The PA-glycans were fractionated according to their sialic acid content as neutral, mono-, and di-sialyl oligosaccharide fractions as indicated.

**Figure 3:**
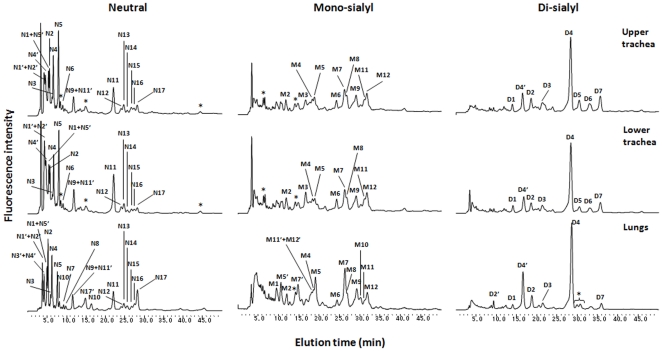
Reverse-phase ODS elution profiles of PA-glycans obtained from three different fractions separated by the DEAE column. The neutral, mono-, and di-sialyl fractions were individually applied onto the ODS column and gave elution profiles according to their hydrophobicity.

**Figure 4:**
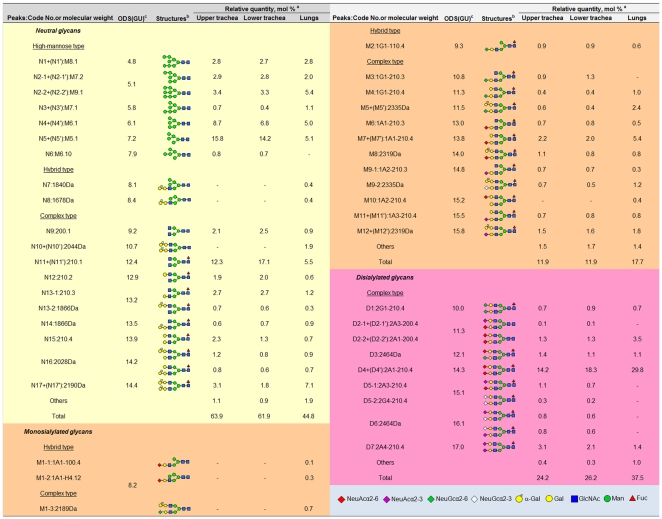
Structures and relative quantities of neutral, mono- and di-sialyl PA-oligosaccharides derived from the porcine upper trachea, lower trachea and lungs of a pig. a, mol % was calculated from the peak area in Figure 3 by comparison with total *N*-glycan content in each porcine tissue. b, Structures of PA-oligosaccharides are represented by symbols as follows: red diamond, NeuAcα2-6; purple diamond, NeuAcα2-3; green diamond, NeuGcα2-6; light blue diamond, NeuGcα2-3; yellow circle with α, α-galactose(Gal); yellow circle, galactose; blue square, N-acetylglucosamine (GlcNAc); green circle, mannose (Man), red triangle, fucose (Fuc). c, Units of glucose (GU) were calculated from the elution times of the peaks obtained from the ODS column in Figure 3.

On the DEAE column, *N*-glycans derived from the upper and lower parts of the porcine trachea and the lungs were separated into four peaks according to their increasing acidicity: neutral (N), monosialyl-NeuAc (M-NeuAc), monosialyl-NeuGc (M-NeuGc) and disialyl (D) glycan fractions with molar ratios (peak areas) of 63.9%, 7.7%, 4.2% and 24.2% from the upper part of the trachea, 61.9%, 7.6%, 4.3% and 26.2% from the lower part of the trachea and 44.8%, 11.0%, 6.7% and 37.5% from the lungs, respectively (Figure 2). Chromatography using the ODS column separated neutral into N1-N17, monosialyl into M1-M12 and disialyl into D1-D7: some were not present in all tissues, but the upper and lower parts of the trachea carried the same *N*-glycans, as shown in Figure 3. These ODS fractions were individually subjected to MALDI-TOF-MS analysis. The N1, N2, N8, N12 and M1 fractions were found to carry two kinds of *N*-glycans and thus were further fractionated by the amide column, resulting in separation of N1 into N5′ and N1, N2 into N2-1 and N2-2, N9 into N9 and N11′, N13 into N13-1 and N13-2, M1 into M1-1, M1-2 and M1-3, M9 into M9-1 and M9-2, D2 into D2-1 and D2-2, and D5 into D5-1 and D5-2 (data not shown). All *N*-glycan structures were identified by the mapping technique on the basis of HPLC elution positions and MALDI-TOF-MS data. Coordinates of all *N*-glycans coincided with those of known references in the GALAXY database except for 14 *N*-glycan fractions including N7, N8, N10, N13-2, N14, N16, N17, M1-3, M5, M8, M9-2, M12, D3 and D6. These fractions containing novel *N*-glycans were subjected to sequential exoglycosidase digestion until the structure changed into a known structure on the map. For example, fraction N17 shown by MALDI-TOF-MS analysis to have a molecular mass of 2190 Da corresponding to (DeoxyHex)1(Hex)7(HexNAc)4PA was resistant to β-galactosidase treatment, suggesting that β-galactose is not its non-reducing terminal residue. Upon α-galactosidase treatment, galactoses were released and gave rise to an oligosaccharide with 13.9 (ODS) and 7.4 (Amide) GUs coinciding with the reference PA-glycan code no. 210.4 in the GALAXY database, Galβ1-4GlcNAcβ1-2Manα1-6(Galβ1-4GlcNAcβ1-2Manα1-3)Manβ1-4GlcNAcβ1-4(Fucα1-6)GlcNAc-PA. The identity of this degalactosylated oligosaccharide was confirmed by co-chromatography and MALDI-TOF-MS analyses. Taking into account the substrate specificity of α-galactosidase, the precursor structure was identified as Galα bi-antennary oligosaccharide Galα-Galβ1-4GlcNAcβ1-2Manα1-6(Galα-Galβ1-4GlcNAcβ1-2Manα1-3)Manβ1-4GlcNAcβ1-4(Fucα1-6)GlcNAc-PA. By the above analyses, a total of 38 *N*-glycan structures of each tissue were revealed and are presented in Figure 4. Total amounts of *N*-glycans derived from delipidated tissues of the porcine upper trachea (311.9 pmol/mg dry delipidated tissue) and lower trachea (404.9 pmol/mg dry delipidated tissue) were 2.3-times and 2.9-times larger than the total amount derived from the porcine lungs (137.7 pmol/mg dry delipidated tissue).

**Figure 5:**
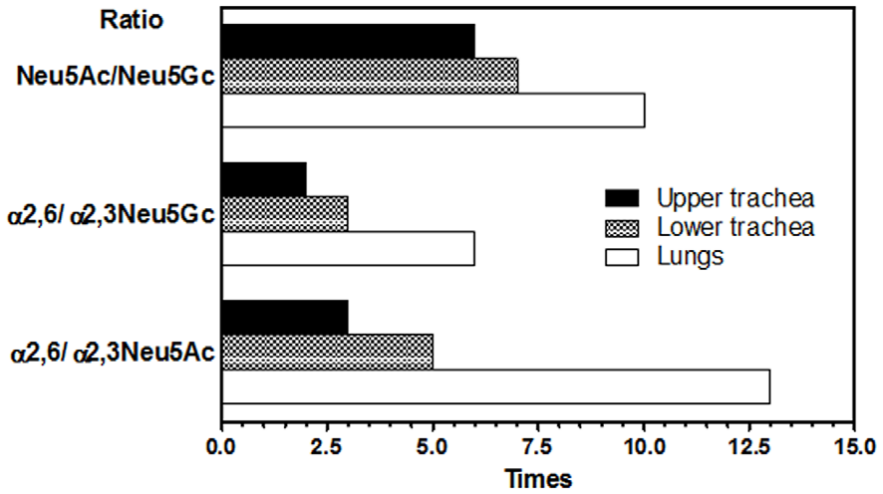
A predominant quantity of Siaα2-6Gal receptor on *N*-glycans in the trachea (upper and lower) and lungs of a pig.

Based on addition of sugar residues to the trimannosyl core, Manα1-6(Manα1-3)Manβ1-4GlcNAcβ1-4GlcNAc, *N*-glycans are classified into three categories: high-mannose, hybrid and complex types (Figure 4). High-mannose-type *N*-glycans in the porcine trachea and lungs contain 2 to 6 α-mannosyl residues added to the trimannosyl core with a bi- or tri-antennary structure without bisecting the GlcNAc residue with no fucose residue at a core region. Hybrid types have a mannosyl residue at one end and an *N*-acetyllactosamine (LacNAc, Gal(β1-4)GlcNAc) unit with Galα or Siaα2-3/2-6 at the other end. Complex type carries GlcNAc and/or LacNAc with Galα or Siaα2-3/2-6 bi-antennary structures. The hybrid and complex types are bi-antennary structures. Sialic acid residues are found at the non-reducing end of *N*-glycans derived from the upper trachea, the lower trachea and the lungs with a molar ratio of 29.1%, 31.2% and 44.7%, respectively. Two main types of sialic acids, 5*-N*-acetylneuraminic acid (NeuAc) and 5-*N*-glycolylneuraminic acid (NeuGc), but not 9-*O*-acetyl-5-*N*-acetylneuraminic acids (9-*O*-Ac-NeuAc), were determined; sialyl glycans possessing NeuAc and NeuGc accounted for 24.8% and 4.3% (lower trachea), 27.1% and 4.1% (upper trachea), and 40.5% and 4.2% (lungs) of total *N*-glycans, respectively, indicating that the upper trachea, lower trachea and lungs expressed NeuAc at 5.8-, 6.6- and 9.6-fold higher levels than the levels of NeuGc, respectively (Figure 2). A disialyl glycan with NeuAcα2-6 on both antennae, NeuAc(α2-6)Gal(β1-4)GlcNAc(β1-2)Man(α1-3)[NeuAc(α2-6)Gal(β1-4)GlcNAc(β1-2)Man(α1-6)]Man(β1-4)GlcNAc(β1-4)[Fuc(α1-6)]GlcNAc, was one of the most abundant in all 38 kinds of *N*-glycans in all tested tissues (14.2%, 18.3% and 29.8% in the upper trachea, lower trachea and lungs, respectively) as indicated in Figure 4. According to outer sugar residues that may serve as receptors for biological and/or pathogenic recognition, the *N*-glycans from all tested tissues were categorized into high-mannose, Galα, Galβ1-4, GlcNAcβ1-2, NeuAcα2-6, NeuAcα2-3, NeuGcα2-6, NeuGcα2-3 with molar ratios as shown in Table 1. Figure 5 shows a comparison of the ratios between different sialic acid species and between different sialyl linkages, which are key determinants of the host range of influenza A viruses, in different tissues. All ratios of NeuAc/NeuGc, NeuAcα2-6/α2-3 and NeuGcα2-6/α2-3 in *N*-glycans were increased towards deep tissues of the porcine respiratory tract. This indicated that human-type Sia NeuAc was expressed more than nonhuman-type Sia NeuGc and that human-type α2-6 linked Sia was expressed more than avain-type α2-3 linked Sia, increasingly from the porcine upper trachea to lower trachea and abundantly in the porcine lung.

**Table 1:** Amounts of α2,3- and α2,6-sialyl receptors in comparison with those of the other outer residues present on *N*-glycans derived from the porcine trachea (upper and lower) and lungs of a pig.

Outer sugar residue on *N*-glycans	Porcine respiratory part, mol %		
	Upper trachea	Lower trachea	Lungs
Mannose	35.6	31.4	22.4
Galα	6.7	4.8	14.2
Galβ1-4	7.3	6.0	6.2
GlcNAcβ1-2	18.5	24.0	8.3
NeuAcα2-6	18.8	22.4	37.6
NeuAcα2-3	6.0	4.7	2.9
NeuGcα2-6	2.8	3.0	3.6
NeuGcα2-3	1.5	1.1	0.6
Unknown	3.0	2.9	4.3

## Discussion

Our results for double lectin immunostaining agree with previous observations that there is dual expression of Siaα2-6Gal (human-type) and Siaα2-3Gal (avian-type) receptors on the surface of porcine tracheal and lung epithelia, while Siaα2-6Gal is dominant [14], [25], [26]. Both human and avian influenza viruses have been isolated from pig populations, indicating that transmission of whole human or avian viruses to swines has occurred in nature [27]. Although SNA and MAA lectins are useful for detection of the localized distribution of α2-6 and α2-3 linkages, respectively, between Sia and Gal, influenza viruses can distinguish not only glycosidic linkages but also types of Sias, which influence the influenza virus host range [25], [28]. HPLC with MALDI-TOF-MS techniques have been continuously developed over the past years for determination of precise *N*-glycan structures and quantitative analysis of those *N*-glycans; influenza virus was reported to bind to but unable to be internalized into cells deficient in *N*-linked glycan, in spite of no deficiency in *O*-linked glycan, suggesting the requirement of *N*-linked glycoprotein for successful endocytosis of influenza virus into its host [18]. By using these techniques, we found that *N*-glycans of the trachea and lungs from a 5-year-old pig were composed of 38 kinds, of which 17, 6, 5 and 10 (tracheal upper and lower parts) and 19, 9, 5 and 5 (lung) were neutral, monosialyl-NeuAc, monosialyl-NeuGc and disialyl, respectively, at neutral/acidic molar ratios of 64∶36 (upper trachea), 62∶38 (lower trachea) and 45∶55 (lung). A few neutral glycans are regarded as virus receptors; however, a triple reassortant H1N1 human isolate, A/Iowa/1/06, has recently been shown to be able to bind to a complex-type *N*-glycan with terminal LacNAc [16]. These neutral *N*-glycans were found in the porcine tracheal upper and lower parts and the porcine lungs with molar ratios of 8.9, 7.4 and 4.1, respectively. In contrast to neutral glycans, Sia with a negative charge is a major receptor and a host range determinant of influenza viruses [25]. Two major entities that influence infectivity of influenza viruses are type and linkage of Sia [1], [29]. Two prevalent Sias (NeuAc and NeuGc) found in mammalian cells were identified, but NeuAc was the predominant form in *N*-glycans of the porcine trachea and lungs (NeuAc:NeuGc, 24.8∶4.3 in the upper trachea, 27.1∶4.1 in the lower trachea, 40.5∶4.2 in the lung). The NeuAc:NeuGc ratio varies among animal species and their tissues, 98∶2 in the duck intestine [28] and NeuGc accounting for more than 90% of Sia in epithelial cells of the horse trachea [25], whereas normal human tissues possess only NeuAc as they have a non-functional hydroxylase to produce NeuGc [30]. The absence of NeuGc may protect humans from infection with some pathogens, such as enterotoxigenic *Escherichia coli* K99 [30], but not from influenza viruses. Overall, both avian and human influenza A viruses appear to exhibit preference for NeuAc rather than NeuGc glycoconjugates. The third not-uncommon Sia, 9-*O*-Ac-NeuAc, a primary receptor determinant of influenza C virus for infection of host cells [31], was not detected, indicating that this *N*-glycan either is not synthesized in the porcine trachea and lung or is present in other tissue. This is in agreement with the fact that influenza C viruses cause mild infection in the upper respiratory tract [32], although the presence of 9-*O*-Ac-NeuAc in the porcine trachea and lungs should not rule out the possibility that *O*-linked glycoproteins or glycolipids carry 9-*O*-Ac-NeuAc.

The type of glycosidic linkage between Sia and Gal on the host cell surface is clearly the principal determinant of the host range restriction of influenza viruses: α2-3-linkage is avian virus preference, while α2-6-linkage is human virus preference. We found that there are gradually increased molar ratios of α2-6-linked sialyl glycans compared to those of α2-3-linked sialyl glycans, 3.2-, 4.9- and 13.2–fold for NeuAc and 1.8-, 2.7- and 5.9-fold for NeuGc in the upper trachea, lower trachea and lungs of the pig, respectively. Our data can explain why influenza viruses, both avian-like [14], [15], classical swine and triple reassortant swine influenza viruses [9], replicated in pigs have changed in their receptor binding preference to human α2-6 receptor and have occasionally been isolated from humans. Our data also provide an explanation of why avian influenza virus before genetic change produced lower virus titers with limitation of spread throughout the porcine respiratory tract in a comparison experiment with a swine virus [33]. Thus, pigs not only serve as ‘mixing vessels’ facilitating reassortment of viral gene segments to produce new influenza strains but also possess abundant proportions of human-type glycan receptors, providing a selective pressure to select/evolve the virus with a receptor preference for this receptor. A selective pressure on the receptor specificity of the viral HA to the human host seems to be a prerequisite for the generation of a virus with pandemic potential in accordance with historical data that most infections by swine viruses cause only limited human-to-human transmission [9]. To become established in a human population, additional factors are likely to be required for optimization of its host-cell tropism. By an integrated biochemical, analytical and data mining approach, it has recently been shown that long α2-6 sialylated glycans with umbrella-like topology (glycans containing multiple repeating lactosamine units, such as NeuAc(α2-6)[Gal(β1-4)GlcNAc (β1-3)]n) may be required for sufficient viral transmission between humans: human-adapted HAs bind with high affinity to umbrella-like topology (long α2-6 glycans), whereas avian and swine HAs preferentially recognize cone-like topology (α2-3 or short α2-6) [29], [34]. The *N*-glycan profile of human bronchial epithelial cells showed the presence of α2-6 long branches [29]. Previous *N*-glycans derived from amniotic membrane (AM) cells of chicken embryonated eggs used for growing viruses isolated from human hosts consist of short α2-6 trisaccharide branches, NeuAc(α2-6)Gal(β1-4)GlcNAc-, but not a long lactosamine structure [35]. Similar to *N*-glycans isolated from AM cells, *N*-glycans with a long branch could not be detected in the porcine trachea and porcine lungs; nevertheless, the possibility that long branches may be present on *O*-linked α2-6 cannot be excluded. However, the recent emergence of S-OIVs with swift human-to-human transmission has confirmed that pigs are the source of the generation of influenza viruses with pandemic potential.

Other glycan modifications, such as fucosylation and sulfation, may be involved in the receptor binding activity of viral HAs [17], [29], [36], [37]. Fucosylation and/or sulfation at Gal or GlcNAc/GalNAc on position 2 or 3, respectively, of the terminal trisaccharide (Sia-Gal-GlcNAc/GalNAc-) appear to affect receptor binding activity of some influenza viruses, such as increase in binding affinity of H5N1 and H7N1 chicken viruses to α2-3-linked Sia [37]. Fucose residue detected in *N*-glycans of the porcine trachea and lungs was found only at the initial GlcNAc of the *N*-glycan core connected to asparagine. Sulfate residue was not detected in porcine trachea and lung *N*-glycans.

It should be noted that glycoconjugate profiles of the porcine respiratory tract, which are involved in mediation and regulation of many physiological and pathological processes, may vary among different species, ages, sex and lifestyle; however, the finding in this study that Siaα2-6Gals are dominant along the respiratory epithelial tract of a 5-year-old female LWD-pig is in agreement with recently reported lectin-binding profiles of 4-8-week-old healthy post-weaned male United Kingdom pigs [26]. All age groups of pigs can be infected by influenza A viruses. During a farrow-to-finish operation, whereas growing-finishing pigs are replaced almost every 6 months for their flesh, mother pigs (sows) remain in a farm for breeding. Influenza infection in a sow herd can cause abortion and can be transmitted to a vulnerable population including their piglets. The changing nature of influenza virus could allow re-infection of sows and, thus, sows could experience several seasons of influenza during their lives and are considered to be involved in the mechanism for maintenance of swine influenza viruses in a farm [38]. Immune evasion from naïve sows results in mutant influenza viruses with antigenic changes and introduction of influenza viruses from other species into this reservoir displaying NeuAcα2-6 predominance may result in generation of new strains that are efficiently transmitted to humans.

In summary, the most striking finding in the present study was the presence of a greater abundance of *N*-glycans carrying α2-6 over α2-3 linkage type, especially di-NeuAc(α2-6) bi-antennary complex type, at the sites of influenza virus replication. This finding explains why influenza viruses that have continuously circulated in pigs displayed an increased affinity for human α2-6 sialylated receptor and indicate the necessity to enhance global surveillance for the emergence of a new variant influenza virus in pigs with the ability to bind to a human-type receptor and the need to establish preparation plans for the next pandemic, not only for H1 and H3 viruses, circulating in swine populations, but also H5 and H7 avian influenza viruses, especially highly pathogenic H5N1 virus experimentally able to infect pigs [39].
